# Echinacoside ameliorates hepatic fibrosis and tumor invasion in rats with thioacetamide-induced hepatocellular carcinoma

**DOI:** 10.17305/bb.2024.10367

**Published:** 2024-10-01

**Authors:** Ajwan Z Albalawi, Areej S Alatawi, Shekha M Al-Atwi, Lama S Alhwyty, Kadi M Alharbi, Shahad A Alshehri, Wasayf A Almarwani, Khulud K Aljohani, Hanan M Hassan, Mohammed M H Al-Gayyar

**Affiliations:** 1 PharmD Program, Faculty of Pharmacy, University of Tabuk, Tabuk, Saudi Arabia; 2 Department of Pharmacology and Biochemistry, Faculty of Pharmacy, Delta University for Science and Technology, Gamasa City, Egypt; 3 Department of Biochemistry, Faculty of Pharmacy, Mansoura University, Mansoura, Egypt; 4 Department of Pharmaceutical Chemistry, Faculty of Pharmacy, University of Tabuk, Tabuk, Saudi Arabia

**Keywords:** β-catenin, cellular communication network factor 2 (CCN2), E-Cadherin, echinacoside, fascin, matrix metalloproteinase-9 (MMP9), mammalian target of rapamycin (mTOR), phosphoinositide 3-kinases (PI3K), platelet-derived growth factor (PDGF)-B, transforming growth factor (TGF)-β1

## Abstract

Hepatocellular carcinoma (HCC) affects approximately 800,000 individuals globally each year. Despite advancements in HCC treatments, there is still a pressing need to identify new drugs that can combat resistance. One potential option is echinacoside, a natural caffeic acid glycoside with antioxidant, anti-inflammatory, antidepressant, and antidiabetic properties. Therefore, we aimed to investigate the ability of echinacoside to exhibit antitumor activity against HCC in rats by ameliorating hepatic fibrosis and tumor invasion. Rats were given thioacetamide to induce HCC, and some were given 30 mg/kg of echinacoside twice a week for 16 weeks. The liver impairment was assessed by measuring serum α-fetoprotein (AFP) and examining liver sections stained with Masson trichrome or anti-TGF-β1 antibodies. The hepatic expression of mRNA and protein levels of transforming growth factor (TGF)-β1, β-catenin, SMAD4, matrix metalloproteinase-9 (MMP9), phosphoinositide 3-kinases (PI3K), mammalian target of rapamycin (mTOR), connective tissue growth factor 2 (CCN2), E-Cadherin, platelet-derived growth factor (PDGF)-B, and fascin were also analyzed. Echinacoside improved the survival rate of rats by decreasing serum AFP and the number of hepatic nodules. Examination of micro-images indicated that echinacoside can reduce fibrosis. It also significantly decreased the expression of TGF-β1, β-catenin, SMAD4, MMP9, PI3K, mTOR, CCN2, PDGF-B, and fascin while enhancing the expression of E-Cadherin. In conclusion, echinacoside exhibits a protective effect against HCC by increasing survival rates and decreasing tumor growth. It also acts as an inhibitor of the hepatic tissue fibrosis pathway by reducing the expression of TGF-β1, β-catenin, SMAD4, PI3K, CCN2, PDGF-B, and mTOR. Additionally, it prevents tumor invasion by suppressing MMP9 and fascin and increasing the expression of E-Cadherin.

## Introduction

Hepatocellular carcinoma (HCC) is considered to be one of the deadliest cancers worldwide. The process of hepatocarcinogenesis is complex, and it involves multiple steps that are affected by various risk factors, leading to tumor metastasis and progression [1]. Despite advances in HCC treatment strategies, patients’ prognosis remains poor due to metastasis, recurrence, and drug resistance. HCC is one of the most commonly diagnosed cancers globally and is ranked as the third leading cause of cancer-related deaths. The number of newly diagnosed HCC cases increases every year. Unfortunately, most patients with HCC greatly suffer due to tumor mutagenesis and progression [2]. Thus, there is an urgent need to research new effective therapeutic agents for managing HCC patients.

Treatment options for liver cancer vary depending on the stage of the cancer, as well as the overall health of the patient. Surgical resection and liver transplantation are options for early-stage liver cancer in patients with cirrhosis. However, there are some relative contraindications for surgical resection in HCC patients, including multiple focal diseases, invasion of a major portal or cirrhosis, and invasion of adjacent segments. Chemotherapy is one of the most important treatment modalities for patients with advanced HCC. Although chemotherapy is an effective cancer treatment, it is not recommended for everyone. Chemotherapy may not be recommended for patients who may experience severe side effects from the treatment [3]. Immunotherapy is a treatment that boosts the immune system to kill cancer cells. It is recommended for patients with advanced cancer and has been shown to increase survival rates and provide long-term cancer control in subsets of patients with HCC. However, patients with autoimmune disorders may be unable to tolerate immunotherapy [4].

Liver fibrosis is greatly associated with HCC. One of the major stimuli of fibrosis is transforming growth factor (TGF)-β. When the signaling pathways of TGF-β are disrupted, it can lead to the development of various human diseases, including liver cancer. The activation of TGF-β is greatly stimulated by overexpression of Wnt/β-catenin, which is also involved in the pathogenesis of fibrotic disorders and HCC. This leads to the phosphorylation and activation of different SMADs, including SMAD4, a protein that plays an essential role in regulating gene expression by acting as a mediator between growth factors and genes inside the cell nucleus [5]. SMAD4 is considered an important mediator of TGF-β signaling and may be a potential therapeutic target for cancer treatment. In addition, TGF-β can produce its effect via a non-SMAD pathway by activating the PI3K/mTOR pathway [6]. Therefore, targeting this pathway may represent a promising therapeutic strategy for treating fibrotic disorders and HCC.

HCC is often associated with macrovascular invasion of the portal vein or hepatic veins in advanced stages. One of the major factors that is associated with tumor invasion is fascin, a protein that helps cells move by bundling actin [7]. The impact of fascin on cell invasiveness involves both alterations in cell motility and matrix protease activity, such as matrix metalloproteinase-9 (MMP9), a protein that breaks down various extracellular matrix (ECM) proteins. It also plays a role in basement membrane degradation and can promote cancer progression. The presence of MMP-9 may indicate a more advanced stage of the disease and a lower chance of survival. In addition, it is considered a promising target for cancer therapy and diagnosis and has been identified as a potential cancer biomarker in several cancer types [8].

Echinacoside is a natural phenol, which belongs to the phenylpropanoid class of caffeic acid glycosides. It is made up of a trisaccharide that consists of two glucose and one rhamnose moieties, linked glycosidically to one caffeic acid and one dihydroxyphenylethanol (hydroxytyrosol) residue at the centrally situated rhamnose. Echinacea is a very beneficial plant, as it has been found to help fight infections and viruses in numerous older studies. Studies also suggest that the antioxidants found in echinacoside could help improve blood sugar levels. Moreover, it alleviates inflammation, chronic pain, and swelling in osteoporosis [9]. Previous studies have reported that echinacoside exhibits antitumor activity against HCC by affecting cell apoptosis and glycolysis [10] or downregulating PI3K/AKT signaling [11]. However, no previous study illustrated the ability of echinacoside to inhibit fibrosis and tumor invasion in HCC.

HCC prevention can be divided into primary, secondary, and tertiary prevention. Primary prevention involves eliminating cancer-predisposing factors through vaccination, lifestyle modifications, and environmental interventions. Secondary prevention aims to delay the progression of chronic liver disease. This approach aims to eliminate the causative agents (HBV and HCV) or hinder the various stages of cancerous progression, such as tumor growth, invasion, and metastasis. Tertiary prevention targets cancer recurrence or de-novo carcinogenesis within 1–2 years after curative treatment [12]. Therefore, we conducted this study to investigate the ability of echinacoside to produce primary protection against HCC in rats via reduction of hepatic fibrosis and tumor invasion. We aimed to investigate the expression of TGF-β1, β-catenin, mammalian target of rapamycin (mTOR), phosphoinositide 3-kinases (PI3K), SMAD4, fascin, MMP9, cellular communication network factor 2 (CCN2), E-Cadherin, and platelet-derived growth factor (PDGF)-B.

## Materials and methods

### Animal models

A total of 40 male Sprague Dawley rats, aged 8–10 weeks old and weighing between 150–200 g, were used in the study. The rats were housed in stainless-steel cages under standard conditions, with a temperature of 25 ± 1 ^o^C and a 12-h light and dark cycle. The rats were divided into four groups, each consisting of ten rats.

Control group: Rats were injected with normal saline for 16 weeks daily.

Echinacoside treated control group: Rats were given 30 mg/kg echinacoside (Santa Cruz Biotechnology Inc., Dallas, Texas) via oral gavage, twice a week for 16 weeks.

HCC group: Rats received a biweekly intraperitoneal injection (ip) of 200 mg/kg thioacetamide in normal saline for a total of 16 weeks.

Echinacoside treatment for the HCC group: Rats received 200 mg/kg thioacetamide via intraperitoneal injection twice a week, along with 30 mg/kg of echinacoside via oral gavage twice a week for 16 weeks.

Prior research on the efficacy of echinacoside for treating HCC has employed varying dosage protocols. For example, one study administered daily intravenous doses of 5 mg/kg over a four-week period [10], while another study used two different intraperitoneal doses (20 and 50 mg/kg) daily for the same duration [11]. Our objective is to explore the potential of oral gavage administration of echinacoside, and we are presently engaged in preliminary investigations in this realm. Testing four different concentrations, 15, 30, 45, and 60 mg/kg, the lowest concentration that produced therapeutic effects was found to be 30 mg/kg.

### Sample collection

The rats were put to sleep using thiopental sodium (40 mg/kg, i.p.). Blood samples were taken from the retro-orbital plexus and spun at 3000 rpm for 5 min. The serum samples were then stored at a temperature of –80 °C. A portion of the liver was separated, sliced, and preserved in a 10% buffered formalin solution for morphological analysis. Another portion of the liver was homogenized in a 10-fold volume phosphate buffer of pH 7.4 and kept at –80 °C.

### Histopathological examination

Hepatic slices were preserved in 10% formalin, embedded in paraffin blocks, and sliced at a thickness of 5 µm. The sections were stained with Masson trichrome to investigate hepatic tissue fibrosis. The sections were coded anonymously and examined in a masked manner. The fibrotic scores were calculated by examining ten fields stained by Masson trichrome in each sample at high power. For immunohistochemistry, the sections were incubated with monoclonal anti-TGF-β and anti-E-Cadherin (MyBioSource, Inc. San Diego, CA, USA) at 4 ^∘^C. The sections were then incubated with a horseradish peroxidase-conjugated antibody. Next, 2% of 3,3′-diaminobenzidine in Tris-buffer was used as a chromogen. Hematoxylin was used as a counterstain [13–15].

### Enzyme-linked immunosorbent assays (ELISA) determination

The concentration of various proteins in homogenized hepatic tissue, such as α-fetoprotein (AFP), MMP9, β-catenin, SMAD4, TGF-β, fascin, mTOR, CCN2, E-Cadherin, PDGF-B, and PI3K were assessed using commercially available ELISA kits. The kits were obtained from USCN Life Science Inc., in Houston, TX, USA for AFP, MMP9, β-catenin, SMAD4, TGF-β, and fascin, and from MyBioSource, Inc., in San Diego, CA, USA for mTOR, CCN2, E-Cadherin, PDGF-B, and PI3K. The manufacturer’s instructions were followed for the assessment of these proteins.

### Quantitative real-time polymerase chain reaction (RT-PCR)

The mRNA levels of *MMP9*, β-catenin, *SMAD4*, *TGF-β*, *mTOR*, *PI3K*, *CCN2*, *PDGF-B*, E-Cadherin, and fascin were analyzed in rat hepatic lysate using our group’s previously described method [16–18]. *GAPDH* was used as a housekeeping gene and internal reference control. The PCR primers used for each gene are listed in Table 1.

**Table 1:** Primer sets used to detect gene expression in rats

**Name**	**Sequence**	**Reference sequence**	
GAPDH	Forward	5′-CCATCAACGACCCCTTCATT-3′	NM_017008.4
	Reverse	5′-CACGACATACTCAGCACCAGC-3′	
TGF-β	Forward	5′-GCTAATGGTGGACCGCAACAAC-3′	NM021578
	Reverse	5′-CAGCAGCCGGTTACCAAG-3′	
β-catenin	Forward	5′-TCCGTCGCCGGTCCACACCC-3′	NM_031144.3
	Reverse	5′-TCACCAACTGGGACGATATG-3′	
SMAD4	Forward	5′-GGATGAAGTCCTGCACACCA-3′	NM_019275.3
	Reverse	5′-GTTGAAGCACTGCCACCTTG-3′	
PI3K	Forward	5′-TTACGGCGGCATGGGAATCT-3′	XM_017595947.2
	Reverse	5′-CCAGCTTTCCCTGAGTGCCT-3′	
mTOR	Forward	5′-CTGCACTTGTTGTTGCCTCC-3′	NM_019906.2
	Reverse	5′-ATCTCCCTGGCTGCTCCTTA-3′	
PDGF-B	Forward	5′-GCCGTGTTCCTTTTCCTCTCT-3′	NM_031524.1
	Reverse	5′-GCACTGCACATTGCGGTTAT-3′	
CCN-2	Forward	5′-ACTGTTGGCGAACAAATGGC-3′	NM_022266.2
	Reverse	5′-CTGCCTCCCAAACCAGTCAT-3′	
MMP9	Forward	5′-CCACCGAGCTATCCACTCAT-3′	NM_031055
	Reverse	5′-GTCCGGTTTCAGCATGTTTT-3′	
Fascin	Forward	5′-TCGACCGGGACACAAGAAAG-3′	NM_001100806.2
	Reverse	5′-GACGCTCTCAGAGTGATCCG-3′	
E-Cadherin	Forward	5′-ATCCTGGCCCTCCTGATTCT-3′	NM_031334.1
	Reverse	5′-CGGGTATCGTCATCTGGTGG-3′	

### Ethical statement

All animal procedures were conducted in accordance with the relevant guidelines and were approved by the Research Ethics Committee of the Faculty of Pharmacy, Delta University for Science and Technology, under the reference number FPDU8/2023.

### Statistical analysis

The results were presented as mean ± SEM. The normality of the distribution of samples in the study was examined using the Kolmogorov–Smirnov test. For a check on rat survival, the Kaplan–Meier procedure was used. For determining significance differences among groups, one-way ANOVA was used followed by the Bonferroni post hoc test. SPSS program (IBM Corp.) version 20 was used to assess the statistical analysis. *P* < 0.05 was considered to indicate a statistically significant difference.

## Results

### Antitumor activity of echinacoside

**Figure 1.**
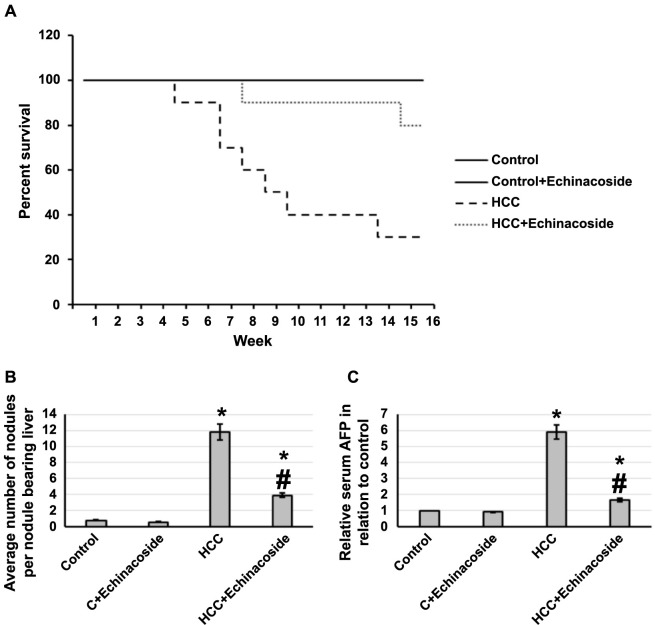
**Effect of HCC and 30 mg/kg echinacoside on rats’ survival (A), the average number of nodules (B), and serum AFP (C).** *Significant difference as compared with the control group at *P* < 0.05. ^#^Significant difference as compared with the HCC group at *P* < 0.05. AFP: Alpha-fetoprotein; C: Control; HCC: Hepatocellular carcinoma.

This study investigated the effects of echinacoside in rats with HCC, revealing a notable increase in the survival rate. About 80% of the rats treated with the drug survived, compared to 30% in the untreated HCC group. The results were accompanied by a decrease in liver nodules and lower serum AFP levels in the echinacoside-treated rats, as depicted in Figure 1.

### Effect of echinacoside and HCC on the hepatic tissue structure

**Figure 2.**
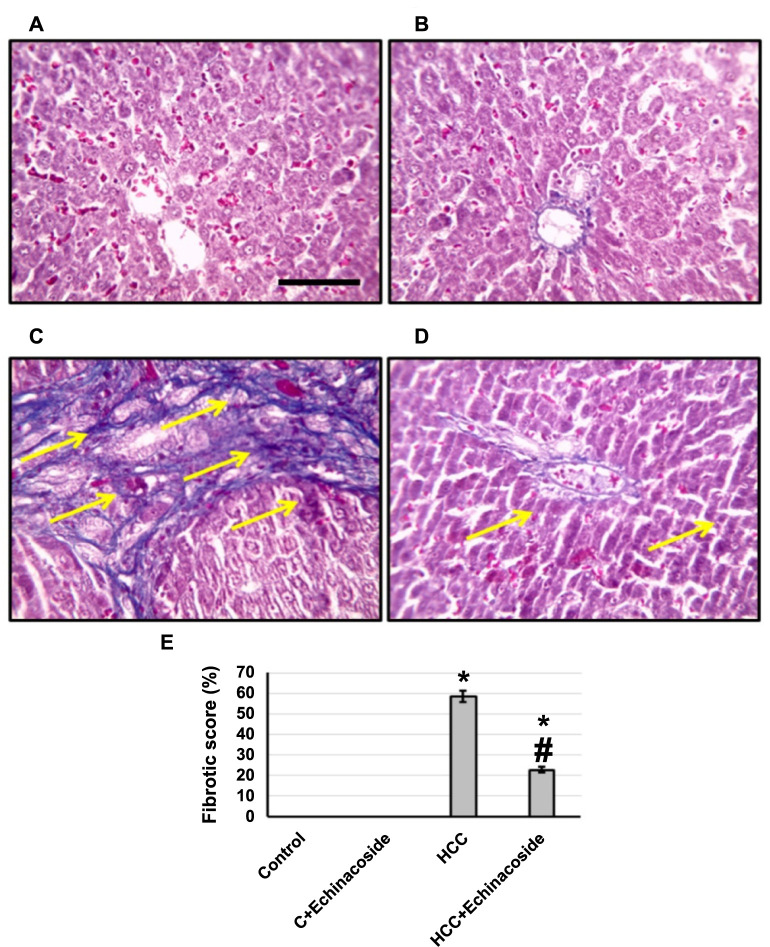
**Hepatic sections stained with Masson trichrome in the control group (A), control group treated with echinacoside (B), HCC (C), and HCC treated with echinacoside (D).** Fibrotic area was determined in 10 fields of high field power and expressed as mean ± standard deviation (E). Yellow arrows indicates areas of fibrosis. *Significant difference as compared with the control group at *P* < 0.05. ^#^Significant difference as compared with the HCC group at *P* < 0.05. Scale bars represent 100 µm. AFP: Alpha-fetoprotein; C: Control; HCC: Hepatocellular carcinoma.

The microsections from the control group, stained with Masson’s trichrome, displayed normal tissue without any notable abnormalities or changes. However, upon examining sections from the HCC group, an increase in fibrotic area was observed. In contrast, sections from HCC rats treated with echinacoside showed a significant improvement in hepatic tissue, with a reduction in the fibrotic area confirmed by statistical analysis of the microsections. These improvements are evident in Figure 2 and may include a decrease in cellular abnormalities or a reduction in the size of cancerous tissues.

### Effect of echinacoside and HCC on the TGF-β expression

**Figure 3.**
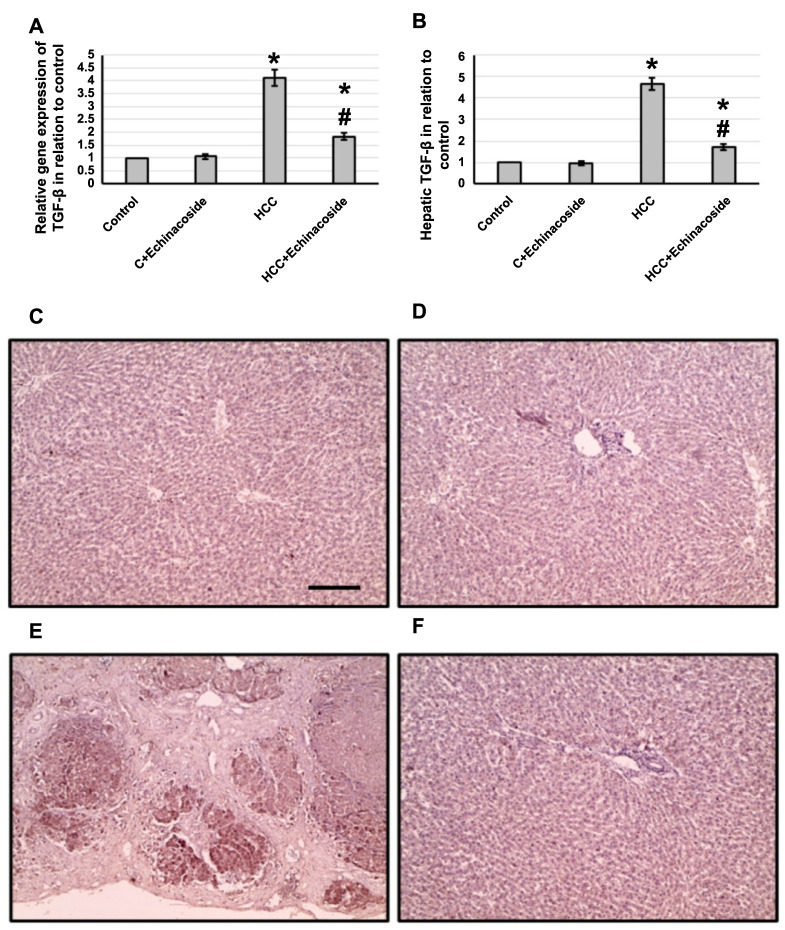
**Effect of HCC and 30 mg/kg echinacoside on hepatic gene expression of *TGF-β* (A) and its hepatic protein level (B).** Hepatic sections stained with anti-TGF-β antibodies in the control group (C), control group treated with echinacoside (D), HCC group (E), and HCC treated with echinacoside (F). *Significant difference as compared with the control group at *P* < 0.05. ^#^Significant difference as compared with the HCC group at *P* < 0.05. Scale bar 100 µm. C: Control; HCC: Hepatocellular carcinoma; TGF-β: Transforming growth factor-β.

We found a 4.12-fold increase in *TGF-β* gene expression, as well as a 4.65-fold increase in hepatic TGF-β protein levels. Staining of the hepatic samples with anti-TGF-β antibodies also indicated a marked increase in the stained areas. However, treatment with echinacoside reversed the effects on the HCC group without impacting the control groups, as demonstrated in Figure 3.

### Effect of echinacoside and HCC on the expression of β-catenin and SMAD4

**Figure 4.**
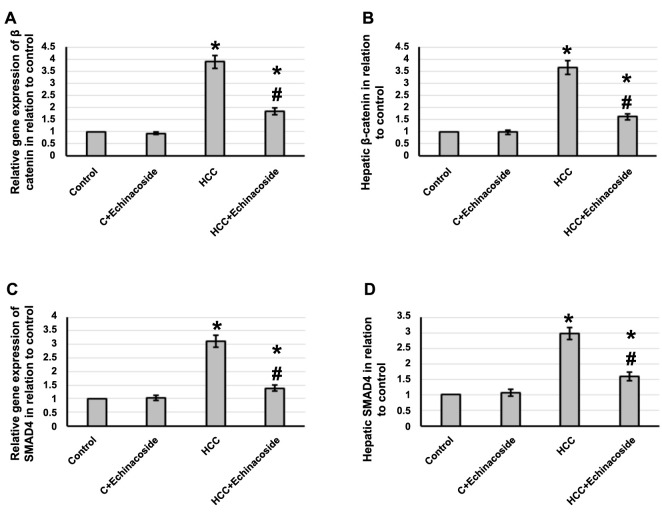
**Effect of HCC and 30 mg/kg echinacoside on hepatic gene expression of β-catenin (A) and *SMAD4* (C) as well as protein expression of β-catenin (B) and SMAD4 (D).** *Significant difference as compared with the control group at *P* < 0.05. ^#^Significant difference as compared with the HCC group at *P* < 0.05. C: Control; HCC: Hepatocellular carcinoma.

The gene expression of β-catenin and *SMAD4* in liver tissues of HCC rats was found to increase significantly by 3.89- and 3.12-fold, respectively, compared to the control group. Furthermore, there was a 3.64- and 3.12-fold increase in β-catenin and SMAD4 levels in hepatic tissue, respectively, compared to the control group. However, in HCC rats, the administration of echinacoside reversed all of these effects without any impact on the control rats. Refer to Figure 4 for further information.

### Effect of echinacoside and HCC on the expression of PI3K and mTOR

**Figure 5.**
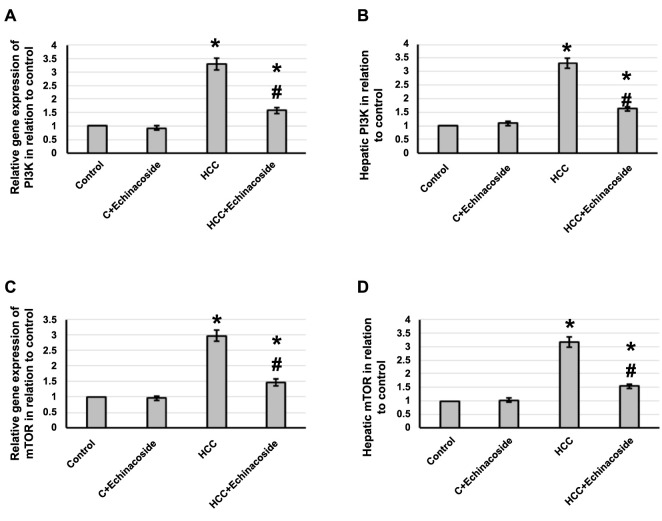
**Effect of HCC and 30 mg/kg echinacoside on hepatic gene expression of *PI3K* (A) and *mTOR* (C) as well as protein expression of PI3K (B) and mTOR (D).** *Significant difference as compared with the control group at *P* < 0.05. ^#^Significant difference as compared with the HCC group at *P* < 0.05. C: Control; HCC: Hepatocellular carcinoma; mTOR: Mammalian target of rapamycin; PI3K: Phosphoinositide 3-kinases.

The examination of liver tissues from rats with HCC showed a notable upregulation in the gene expression of *PI3K* and *mTOR*, specifically by 3.08- and 3.28-fold, respectively. Additionally, there was a significant increase in the levels of PI3K and mTOR in the HCC rat liver tissues, with a 3.49- and 3.32-fold change compared to the control group. However, the administration of echinacoside reversed these effects in HCC rats without affecting the control group. For more information, refer to Figure 5.

### Effect of echinacoside and HCC on the expression of CCN2 and PDGF-B

**Figure 6.**
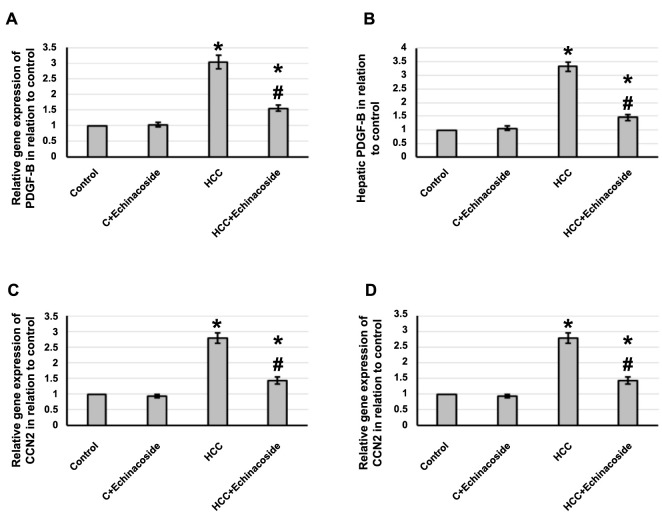
**Effect of HCC and 30 mg/kg echinacoside on hepatic gene expression of *PDGF-B* (A) and *CCN2* (C) as well as protein expression of PDGF-B (B) and CCN2 (D).** *Significant difference as compared with the control group at *P* < 0.05. ^#^Significant difference as compared with the HCC group at *P* < 0.05. C: Control; HCC: Hepatocellular carcinoma; PDGF-B: Platelet-derived growth factor-B; CCN2: Cellular communication network factor 2.

The gene expression of *PDGF-B* and *CCN2* in liver tissues of rats with HCC was significantly increased by 3.04- and 2.79-fold, respectively. Similarly, levels of PDGF-B and CCN2 in hepatic tissue were elevated by 3.32- and 2.91-fold, respectively, compared to the control group. However, treatment with echinacoside reversed these effects in HCC rats, without affecting control rats. For more details, refer to Figure 6.

### Effect of echinacoside and HCC on the expression of MMP9 and fascin

**Figure 7.**
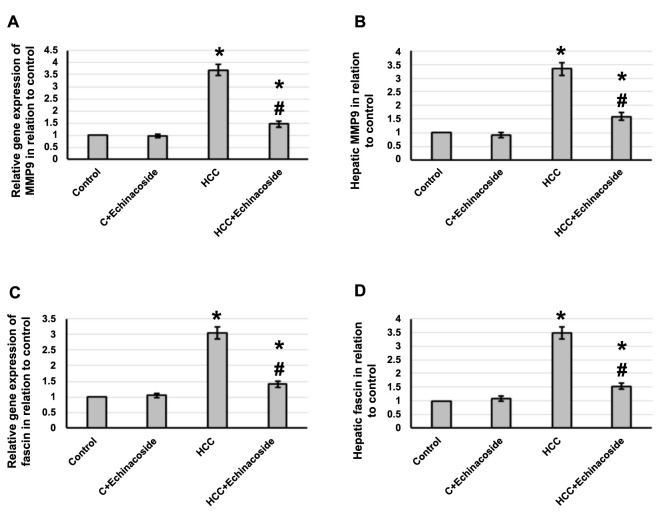
**Effect of HCC and 30 mg/kg echinacoside on hepatic gene expression of *MMP9* (A) and fascin (C) as well as protein expression of MMP9 (B) and fascin (D).** *Significant difference as compared with the control group at *P* < 0.05. ^#^Significant difference as compared with the HCC group at *P* < 0.05. C: Control; HCC: Hepatocellular carcinoma; MMP9: Matrix metalloproteinase 9.

Hepatic tissue analysis in HCC rats showed elevated gene expression of MMP9 and fascin by 3.69- and 3.04-fold, respectively. In addition, MMP9 and fascin levels in hepatic tissue were 3.34- and 3.49-fold higher in HCC rats compared to the control group. Echinacoside counteracted these changes in HCC rats while not affecting the control rats. See Figure 7 for further details.

### Effect of echinacoside and HCC on the expression of E-Cadherin

**Figure 8.**
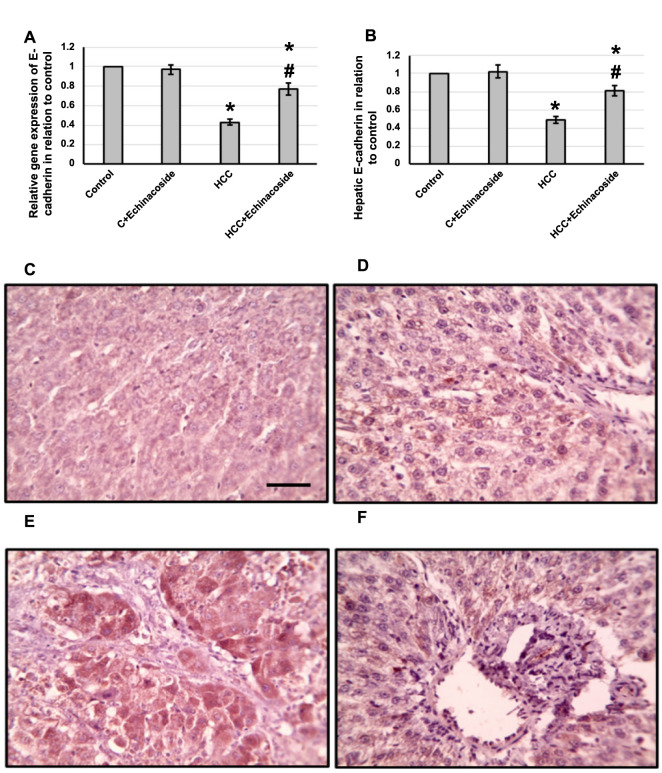
**Effect of HCC and 30 mg/kg echinacoside on hepatic gene expression of E-Cadherin (A) and its hepatic protein level (B).** Hepatic sections stained with anti-E-Cadherin antibodies in the control group (C), control group treated with echinacoside (D), HCC group (E), and HCC treated with echinacoside (F). *Significant difference as compared with the control group at *P* < 0.05. ^#^Significant difference as compared with the HCC group at *P* < 0.05. Scale bar 100 µm. C: Control; HCC: Hepatocellular carcinoma.

We noticed a significant decrease in the gene expression of E-Cadherin by 57%. Moreover, the levels of E-Cadherin protein in the liver decreased by 51%. The liver sections, which were stained with anti-E-Cadherin antibodies, also showed a noticeable decrease in the stained areas. However, when echinacoside was administered, the negative effects on the HCC group were reversed, without affecting the control groups. This is shown in Figure 8.

## Discussion

HCC is a common type of cancer that ranks sixth in terms of prevalence and fourth in terms of cancer-related deaths worldwide with a five-year survival rate of approximately 18%. The physical and emotional changes caused by chemotherapy can significantly affect the quality of life of patients. However, despite its effectiveness, chemotherapy can also lead to serious side effects and is expensive [19]. To test potential anti-tumor compounds, we induced HCC in rats using thioacetamide. The results showed a significant reduction in the survival rate, with only 30% of the rats surviving. Liver nodules and serum AFP levels also increased, and Masson trichrome-stained microsections showed an expansion of fibrotic tissue in the HCC group. In parallel, echinacoside is a naturally active constituent with numerous important pharmacological activities, such as alleviating inflammation, chronic pain, and swelling in osteoporosis. Our study found that treating HCC rats with echinacoside resulted in a significant increase in their survival rate and a significant reduction in the number of nodules and serum AFP. Furthermore, the examination of micro-sections from HCC rats treated with echinacoside and stained with Masson trichrome showed a marked improvement in hepatic tissue and reduced the fibrosis area.

TGF-β is a group of multifunctional polypeptides secreted by various cells, such as monocytes, T cells, or blood platelets. These polypeptides have diverse effects on the division and activity of cells, such as regulating cell proliferation, differentiation, and apoptosis. When TGF-β family proteins bind to their cell surface receptors, they activate intracellular signaling pathways, including SMADs, to induce changes in gene expression. These changes can lead to the activation or repression of target genes, resulting in a wide range of cellular responses [5]. However, when their signaling pathways are disrupted, it can lead to the development of various human diseases. In the liver, TGF-β signaling is involved in all stages of disease progression, from initial liver injury to HCC. TGF-β plays a dual role in the malignant cell during liver cancer development. It acts as a suppressor factor in the early stages but contributes to later tumor progression once cells escape its cytostatic effects [20]. Additionally, TGF-β can affect the response of the cells that make up the tumor microenvironment, which can also contribute to HCC progression and drive immune evasion of cancer cells [21]. Therefore, targeting the TGF-β pathway could be an effective therapeutic option for HCC treatment. However, we found a significant elevation in the expression of TGF-β in HCC rats, which was inhibited by echinacoside treatment. Echinacoside was reported previously to inhibit the expression of TGF-β in LPS-induced apoptosis and inflammation in intestinal epithelial cells [22], kidney apoptosis model [23], and dextran sulfate-induced colitis [24]. However, no previous study illustrated the ability of echinacoside to reduce the expression of TGF-β in HCC.

β-catenin is a key component of the Wnt/β-catenin signaling pathway, which plays a crucial role in coordinating cell–cell adhesion and gene transcription. This pathway involves almost every aspect of embryonic development, including cell proliferation, differentiation, and migration. In addition, it controls homeostatic self-renewal in several adult tissues, such as the intestine, skin, and liver [25]. Recent research has shed light on the role of the Wnt/β-catenin pathway in the pathogenesis of fibrotic disorders. Organ fibrosis is a common feature of many chronic diseases, such as liver cirrhosis, pulmonary fibrosis, and kidney fibrosis. It is characterized by the excessive accumulation of ECM proteins, which disrupts tissue architecture and impairs organ function. Sustained reactivation of the Wnt/β-catenin pathway has been shown to promote fibroblast activation, myofibroblast differentiation, and ECM deposition, all of which contribute to the development of fibrosis [26]. Therefore, targeting this pathway may represent a promising therapeutic strategy for treating fibrotic disorders. Deregulated β-catenin signaling is one of the main genetic alterations in HCC. HCCs activating the β-catenin pathway have unique gene expression patterns and pathological features. The activated β-catenin pathway works with multiple signaling cascades to drive HCC formation and functions through its downstream effectors [27]. The deregulated β-catenin pathway in HCC has been shown to impact the disease’s development and progression significantly. It involves various cellular processes, including cell proliferation, differentiation, and apoptosis. The pathway also interacts with other signaling pathways, such as the PI3K/Akt and MAPK/ERK pathways, to promote HCC formation and progression [28]. However, we found a significant elevation in the expression of β-catenin in HCC rats, which was inhibited by echinacoside treatment. Echinacoside was reported previously to inhibit the expression of β-catenin in breast cancer [29], but no previous study illustrated the ability of echinacoside to downregulate β-catenin in HCC.

SMAD4 is a protein essential for adequately functioning the SMAD pathway, a signaling pathway regulating various cellular processes, such as cell growth, differentiation, and apoptosis. It acts as a mediator between growth factors from the TGF-β family outside the cell and genes inside the cell nucleus, thereby playing a crucial role in regulating gene expression. SMAD4 is a highly conserved protein found in all metazoans, including humans, and is encoded by the SMAD4 gene [30]. It also plays a critical role in developing and maintaining various organs and tissues, including the liver, pancreas, and gastrointestinal tract. Mutations in the SMAD4 gene have been associated with various diseases, including pancreatic cancer, juvenile polyposis syndrome, and hereditary hemorrhagic telangiectasia [31]. SMAD4 is the center of the TGF-β pathway and is considered an essential mediator of TGF-β signaling, which is overexpressed in various types of cancer. Interestingly, deleting the SMAD4 gene has been found to have protective effects against pancreatic cancer [32]. This suggests that SMAD4 may be a potential therapeutic target for cancer treatment. However, we found a significant elevation in the expression of SMAD4 in HCC rats, which was inhibited by echinacoside treatment. However, no previous study illustrated the ability of echinacoside to downregulate SMAD4 in any disease model.

Our research involved a thorough analysis of the PI3K/mTOR pathway, which plays a crucial role in the survival of tumor cells, resistance to apoptosis, and angiogenesis. This pathway can be activated either directly through elevated levels of ROS and VEGF or indirectly through the release of histamine [33]. We focused on the function of mTOR, which is responsible for autophagy inhibition and enhances the signaling pathways of MAPK and AKT [34]. Inhibiting PI3K and mTOR has shown promising results in combating tumor cells [35]. Our study found that echinacoside effectively decreased the expression of both PI3K and mTOR in HCC rats. This is a significant finding as previous studies have shown that echinacoside directly inhibits the PI3K/mTOR pathway in different cancer types, such as HCC [11], breast cancer [36], Ehrlich carcinoma [37], colorectal cancer [38], and ovarian cancer [39]. Overall, our research provides valuable insights into the potential of echinacoside as a therapeutic agent for breast cancer treatment. By inhibiting the PI3K/mTOR pathway, echinacoside could effectively combat tumor cells and improve the outcomes.

The connective tissue growth factor, also known as CCN2, is a member of the CCN family of matricellular proteins that includes CCN1-6. These proteins are cysteine-rich and contain an N-terminal secretory peptide, as well as four multi-functional domains that can interact with a wide range of binding partners. CCN2’s second domain facilitates its interaction with TGF-β, while the third domain binds to MMPs. The final C-terminal motif allows it to interact with PDGF [40]. The protein known as PDGF is a member of the pro-inflammatory growth factor family and has been found to be essential in recruiting pericytes. Its signaling pathway has been linked to the development of HCC, and numerous studies have demonstrated its crucial role as a pathogenic factor in various solid tumors. Evidence suggests that the PDGF family also plays a fundamental part in liver cirrhosis, which is a significant risk factor for the onset of HCC [41]. We found a significant increase in the expression of both CCN2 and PDGF in HCC rats that was reversed by echinacoside treatment. However, no previous study illustrates the ability of echinacoside to reduce the expression of CCN2 and PDGF in any animal model.

MMP-9 is a matrix metalloproteinase extensively studied due to its crucial role in tumor metastasis and cancer cell invasion. It is responsible for breaking down various ECM proteins, including collagen, laminin, and fibronectin, which help regulate the remodeling of ECM. MMP-9 plays a role in basement membrane degradation since the basement membrane contains collagens, including Type IV Collagen, which can be degraded by MMP-9 [8]. Moreover, MMP-9 can also cleave many plasma surface proteins, such as cytokines, growth factors, and adhesion molecules, releasing them from the cell surface and modulating their biological activity. This process can further promote cancer progression by enhancing angiogenesis, immune evasion, and tumor cell survival [42]. Therefore, MMP-9 is considered a promising target for cancer therapy and diagnosis. MMP-9 has been identified as a potential cancer biomarker in several cancer types due to its critical role in the invasive potential of tumors. Moreover, MMP-9 has been used to predict tumor recurrence and survival in patients with HCC. Studies have shown that MMP-9 is superior to MMP-2 for tumor recurrence and survival prediction in HCC patients. The serum MMP-9/MMP-2 ratios in patients with HCC were significantly higher than those of other groups, indicating that the serum MMP-9/MMP-2 ratio is a potential biomarker of HCC [43]. Our study found that echinacoside effectively decreased the expression of MMP9 in HCC rats. Echinacoside was found previously to reduce the expression of MMP9 in endometrial cancer [44], however, no previous study illustrates the ability of echinacoside to reduce the expression of MMP9 in HCC.

E-cadherin is a transmembrane protein that plays a crucial role in forming stable adherent junctions. It is associated with dedifferentiation, infiltration, and metastasis in many types of cancer [20]. It is reversely correlated with fascin, a protein that plays a crucial role in forming filopodia and lamellipodia, which are membrane protrusions that help cells move. It does so by bundling actin, a protein that forms the structural framework of cells. Fascin expression has been linked to the prognosis and progression of various neoplasms [7]. Recent studies suggest that fascin may serve as a novel marker of progression in HCC patients and a significant indicator of poor prognosis [45]. This means that the presence of fascin in HCC patients may indicate a more advanced stage of the disease and a lower chance of survival. Our study found that echinacoside effectively decreased fascin and increased E-Cadherin expression in HCC rats. However, no previous study illustrated the ability of echinacoside to affect the expression of fascin and E-Cadherin in HCC.

It is crucial to understand that the liver utilizes the cytochrome P450 (CYP450) 2E1 enzyme to process thioacetamide, leading to the formation of thioacetamide-S-oxide and thioacetamide-S-dioxide. The latter triggers oxidative stress by initiating lipid peroxidation of the hepatocellular membrane [46]. CYPs are responsible for the breakdown of xenobiotics, which include carcinogens, steroids, and drugs. They participate in approximately 75% of enzyme reactions involved in drug metabolism. Echinacoside works by inhibiting CYPs, which is a significant mechanism [47]. Therefore, it is essential to exercise caution when taking echinacoside or herbal remedies containing echinacoside concurrently with other drugs that share the same CYPs, particularly those with a narrow therapeutic window or known adverse effects that require further future studies.

It is important to note that there are some limitations to the research findings. Firstly, the study followed the preventive mode instead of the treatment mode. This means that further studies are needed to evaluate the possibility of using echinacoside in a therapeutic approach. Secondly, rats were used as a model in the study. However, rats have different metabolic pathways and drug metabolites than humans, which can lead to different dosing and various ways of the body dealing with the drugs. Therefore, the results obtained from this study should be interpreted with caution when extrapolating to humans. Additionally, it is crucial to note that there are many methods for tumor induction in rats, but for this study, only the chemical induction of HCC in rats using thioacetamide was used. Despite these limitations, the study provides valuable insight into the potential antitumor effects of echinacoside and could serve as a basis for future investigations in humans.

## Conclusion

Echinacoside, a naturally occurring compound found in various plants, possesses potent anti-cancer properties and may aid in preventing the development of HCC. Studies have demonstrated that Echinacoside increases the survival time of rats and significantly reduces the number of nodules and serum AFP. It also improves the structure of hepatocytes and decreases fibrosis. Further investigation into the molecular mechanisms reveals that it inhibits the pathway of hepatic tissue fibrosis by decreasing the expression of several key proteins, including TGF-β1, β-catenin, SMAD4, PI3K, mTOR, CCN2, and PDGF-B. Furthermore, Echinacoside has been found to suppress the expression of MMP9 and fascin, two proteins that play a critical role in tumor invasion and metastasis, while increasing the expression of E-Cadherin. These effects suggest the potential of Echinacoside in preventing the spread of cancer cells to other parts of the body and improving the overall prognosis of HCC. Additionally, Echinacoside exhibits promise in treating liver fibrosis.

## Data Availability

Data are available from the corresponding author upon suitable request.
